# Tregs in pregnancy with type 1 diabetes mellitus: pilot study

**DOI:** 10.1590/1806-9282.20241283

**Published:** 2025-03-17

**Authors:** Juliana Malafaia Von, Rosiane Mattar, Erika Ono, Evelyn Traina, Karen Priscilla Tezotto Pendeloski, Patricia Medici Dualib, Edward Araujo, Silvia Daher

**Affiliations:** 1 Universidade Federal de São Paulo, Escola Paulista de Medicina, Department of Obstetrics – São Paulo (SP), Brazil.; 2 Universidade Federal de São Paulo, Escola Paulista de Medicina, Department of Medicine, Discipline of Endocrinology – São Paulo (SP), Brazil.

**Keywords:** Pregnancy, Regulatory T lymphocytes, T-lymphocyte subsets, Type 1 diabetes mellitus

## Abstract

**OBJECTIVE:**

Pregnancy in women with type 1 diabetes mellitus has been associated with adverse outcomes due to persistent hyperglycemia and impaired maternal–fetal interactions. Regulatory T cells seem to exert a critical role in this process. Pregnancy can change the profile of Treg cells and affect the outcome of pregnancy; therefore, our purpose was to characterize the profile of regulatory T cells in the peripheral blood of pregnant and nonpregnant (controls) women with type 1 diabetes mellitus.

**METHODS:**

This prospective case-control study recruited 36 women with type 1 diabetes mellitus, 16 pregnant and 20 nonpregnant. Peripheral blood samples were collected in the three trimesters of pregnancy and only once in the control group. Flow cytometry was used to assess peripheral blood T subpopulations: CD3+, CD4+, CD8+, CD4+ Treg (CD4+CD25+CD127-FOXP3+), and CD8+ Treg (CD8+CD25+FOXP3). In addition, the expression of CD4+CD25high and CD4+CD25low was analyzed.

**RESULTS:**

Compared to controls, the pregnant women (regardless of the trimester) presented a lower percentage of TCD4+CD25high, TCD4+CD25low, and CD8 Treg (CD8+CD25+FOXP3+). Moreover, a higher percentage of total TCD8+ lymphocytes was observed in pregnant women than in controls.

**CONCLUSION:**

This study reported changes in the circulating Treg cell profile that seem to be associated with pregnancy in type 1 diabetes mellitus patients and pregnancy outcomes.

## INTRODUCTION

Type 1 diabetes mellitus (T1DM) is an autoimmune disease characterized by the destruction of insulin-producing beta (β) cells in the pancreatic islets, leading to insulin deficiency and severe alterations in homeostasis^1^. Until a few decades ago, women with T1DM were not encouraged to get pregnant due to the high incidence of adverse outcomes. With the advance in medical care and consequent improvement of glycemic control, pregnancy became possible in these women, but it is still a risky condition^2,3^. Besides tight glycemic control, an adequate maternal immune response to the fetus is critical for the development of a successful gestation^4^.

Among the various immune components involved in maternal and fetal interactions, T regulatory (Treg) seems to play a central role. Most of these cells are CD4+ lymphocytes, defined by the expression of the CD25 receptor on its surface, as well as by the intracellular marker FOXP3. In addition to these parameters, the expression of markers such as CD127 is low or completely absent^5^. Based on new investigations, it has been described that CD4+Treg lymphocytes can be divided into two distinct subpopulations: according to the intensity of CD25 expression, high and low, which characterizes a high or low suppressor function, respectively^6^. In addition, there is a CD8+Treg population, which is characterized by the expression of CD8+CD25+FOXP3+. The functions of these cells are not fully understood. Nevertheless, it has been suggested that these lymphocytes have a suppressor profile^7^.

Other markers can be used to functionally characterize the Treg cell group such as CTLA4, CD45RA, Helios, and the intensity of expression of FOXP3. The Treg cells can be divided into subgroups as naïve Treg cells CD25+CD45RA+FOXP3low, effector Treg cells as CD25highCD45RA-FOXP3high, and non-suppressive CD4+T cells as CD25+CD45RA-FOXP3low^8,9^.

In line, different investigators have reported alterations (function/number) of Treg cells in individuals with T1DM. Studies have reported changes in the Treg profile in individuals with T1DM, such as increased frequency of Tregs producing proinflammatory cytokines^10^, and decreased IL-2 sensitivity^11^.

The objective of this study was to evaluate the profile of T lymphocytes, especially Treg, in pregnant and nonpregnant (controls) women with T1DM.

## METHODS

This prospective case-control study recruited women with T1DM in the Diabetes and Pregnancy and in the Endocrinology Antenatal Care Clinics of Paulista School of Medicine—Federal University of São Paulo (EPM-UNIFESP) between 2020 and 2022. This study was approved by the Ethics Committee of UNIFESP (#30198220.5.0000.5505). Informed consent was obtained from all participants.

The study included 36 T1DM women: 16 pregnant and 20 nonpregnant. The diagnosis of T1DM was established according to the parameters recommended by the American Diabetes Association (ADA): fasting glucose ≥126 mg/dL or plasma glucose after 2 h of oral glucose tolerance test ≥200 mg/dL or A1C (Glycated hemoglobin) ≥6.5% or if the patient has classic symptoms of hyperglycemia or hyperglycemic episodes with random plasma glucose ≥200 mg/dL and being on insulin therapy. All participants had a previous diagnosis of at least 5 years of the disease, were aged between 18 and 40 years, and had body mass index (BMI) between 18.5 and 35 kg/m^2^ when recruited. For the pregnant group, singleton pregnancy with a live fetus without congenital anomalies was required. Exclusion criteria for both groups were as follows: women with other types of diabetes or other chronic systemic diseases, with any type of active infection, history of solid organ transplant or using antibiotics, immunosuppressants, antihistamines, or anti-inflammatory drugs at the time of the sample collection.

Blood samples were obtained by venipuncture into a 4-mL EDTA tube (BD Diagnostics, USA). Among the 16 pregnant women, we collected samples at all three trimesters from five patients, at two trimesters (first and second or second and third) from four women, and at only one trimester from seven participants (first or second or third). We also collected one single sample from 20 nonpregnant T1DM women. The samples were analyzed within 1 h after collection. Cell subsets were determined by flow cytometry. All samples were evaluated in the same FACSCantoTM cytometer (BD Biosciences, San Jose, CA, USA), and data were analyzed using the FlowJo software (Tree Star, Inc.). We used the parameters obtained with the Fluorescence Minus One (FMO) as controls. The data were presented as median, percentages, and standard deviations.

**Figure 1:**
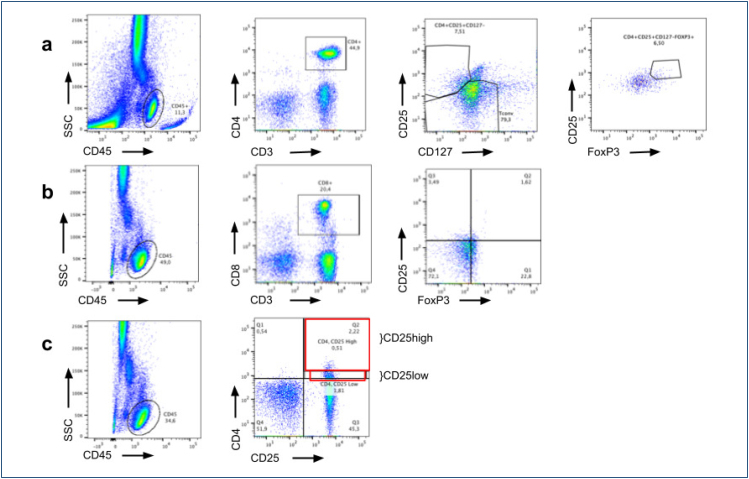
Representative FACS profile of human T lymphocytes. (A) Gating strategy of CD45+ T lymphocytes of which CD3+CD4+ were gated then CD3+CD4+CD25+CD127– and then we gated of which were FOXP3+ to characterize CD4+ Treg cells. (B) Gating strategy of T lymphocytes in an FSC/SSC density plot in the total acquired events then CD3+CD8+ were gated and then we gated the CD25+FOXP3+ to characterize CD8+ Treg cells. (C) Gating strategy of CD45+ T lymphocytes and then CD4+CD25+ divided by the intensity of CD25+ with high and low.

In a tube for cytometry, 100 μL of peripheral blood was added together with the following antibodies with the amount described by the manufacturer: anti-CD3 (APC-Cy7), anti-CD45 (V500), anti-CD4 (Alexa Fluor 647), anti-CD8 (BV421), anti-CD25 (PerCP 5.5), and anti-CD127 (BB515). The tubes were then incubated for 15 min in the dark at room temperature. After incubation, 2 mL of red blood cell lysis solution (BD Biosciences, San Jose, CA, USA), previously diluted with 200 μL of Facs Lysing in 1,800 μL of deionized water, was added. The final solution was homogenized, and the tubes were left in the dark at room temperature for 15 min. Then, the tubes were centrifuged at 800×*g* for 5 min at 4°C. After centrifugation, the cells were fixed and permeabilized using the fixation/permeabilization kit (eBioscience Inc, San Diego, CA, USA) according to the manufacturer's instructions. After processing, anti-FOXP3 [preeclampsia (PE)] monoclonal antibody was added. The material was incubated for 30 min at 4°C in the dark. Then, 2 mL of MACS buffer solution [Phosphate-buffered saline (PBS) 1× (Dulbecco s/Ca s/MG—Cutilab, Brazil) containing 0.1% alumina with bovine serum (BSA—Sigma-Aldrich Corp., St. Louis, MO, USA) and 2 mL EDTA (Sigma-Aldrich Corp, St. Louis, MO, USA)] was added. The sample was centrifuged again at 800×*g* for 5 min at 4°C. Then, the supernatant was discarded, and the cell pellet was resuspended in 500 μL of PBS supplemented with bovine albumin for evaluation on the BD LSRFortessa cytometer (BD Biosciences, San Jose, CA, USA), as described in Figure 1.

The statistical analyses were performed using the statistical software JASP Version 0.16.2 (University of Amsterdam, 2022). Continuous numeric variables are presented as median with minimum and maximum, and categorical variables are represented as frequency and percentages (%). Values of p<0.05 were considered statistically significant and are marked with an asterisk.

## RESULTS

The main characteristics of participants are presented in Table 1. There was a significant difference regarding the A1C level, which was higher in the control group. We also observed that the use of the drug acetylsalicylic acid (Aspirin) was greater in pregnant women than in the control group.

**Table 1:** Clinical features of patients with type 1 diabetes mellitus, controls (nonpregnant), and pregnant women.

		Controls n=20	Pregnant women n=16	p
Age (years)^1^		25.7 (±5.7)	27.4 (±4.4)	0.308
BMI (pregestational for pregnancy) (kg/m^2^)^1^		25.0 (±4.3)	25.4 (±3.8)	0.792
Previous pregnancies^1^		0.5 (±0.7)	0.8 (±1.0)	0.270
Race (%)^2^				
	White	60%	43.7¨%	0.619
	Black	10%	12.5%	
	Mixed	30%	43.7%	
T1DM duration (years)		15.9 (±7.8)	15.9 (±5.8)	0.987
A1c (pregestational				
	for pregnancy) (%)^1^	8.8 (±1.7)	7.7 (±1.4)	0.040*
Insulinization method^2^				
	Syringe	10%	25%	0.434
	Insulin pen	60%	56.3%	
	Insulin pump	30%	18.7%	
Aspirin^2^				
	No	0%	43.7%	<0.001*
	Yes	100%	56.3%	

A1c: glycated hemoglobin; BMI: body mass index; T1DM: type 1 diabetes mellitus.

1 Student-t test;

2 Fisher's exact test.

* p<0.05.

Only one pregnant woman developed an obstetric disorder (PE). All children were born alive: 6 were large for gestational age, 9 neonates exhibited hypoglycemia, and 13 presented with jaundice.

Considering all T-cell subsets, there were no significant differences between the gestational trimesters. In addition, no difference was detected in the frequency of circulating CD3+ and CD4+T lymphocytes of pregnant women in comparison to nonpregnant patients. However, we observed higher expression of CD8+ in pregnant women. In addition, we identified lower percentages of TCD4+CD25high, TCD4+CD25low, and Treg CD8 cells (CD8+CD25+FOXP3+) in the subgroups of pregnant women compared to controls (Table 2).

**Table 2:** Immunophenotypic characteristics of the percentages of TCD3+, TCD4+, TCD8+, Treg cells, and their subgroups in the peripheral blood in the group of women with type 1 diabetes mellitus controls (nonpregnant) and pregnant women divided by trimesters.

	Control	Pregnant women			p
	n=20	First trimester n=7	Second trimester n=12	Third trimester n=11	
CD3+	76.0 (63.0–84.7)	77.3 (70.5–88.0)	77.7 (69.6–89.5)	78.6 (70.7–90.1)	0.522
CD4+	44.7 (26.4–54.3)	41.4 (32.8–53.5)	43.7 (36.8–51.5)	42.1 (35.8–54.1)	0.736
CD8+	20.4 (10.1–29.5)	31.40 (20.0–37.3)	22.7 (18.5–36.3)	24.0 (17.4–30.9)	0.006a*; 0.027b*; 0.017c*
CD4+CD25 High	0.28 (0.05–0.5)	0.03 (0.0–0.8)	0.03 (0.01–0.5)	0.07 (0.01–0.2)	0.008a*; 0.006b*; 0.002c*
CD4+CD25 Low	1.00 (0.2–2.5)	0.2 (0.1–1.7)	0.1 (0.03–2.1)	0.2 (0.1–1.5)	0.007a*; 0.001b*; 0.017c*
CD4+CD25+CD127-FOXP3+	5.5 (3.0–8.7)	5.3 (3.4–6.5)	5.4 (3.4–8.8)	5.3 (2.8–8.5)	0.581
CD8+CD25+FOXP3+	1.3 (0.4–2.4)	0.4 (0.1–1.2)	0.3 (0.1–0.7)	0.3 (0.2–0.8)	0.002a*; <0.001b,c*

Data obtained with the Kruskal-Wallis test and Dunn test: a—Control versus pregnant first trimester; b—control versus pregnant second trimester; c—control versus pregnant third trimester. Data are presented as median, minimum, and maximum.

* p<0.05.

## DISCUSSION

This pilot study showed a lower number of some T-cell subsets with a suppressive profile in T1DM pregnant women, regardless of the trimester, compared to nonpregnant patients with the same pathology. We evaluated different subpopulations of T lymphocytes characterized by membrane and intracellular markers, which distinguish functionally distinct cell populations. The two groups of participants were matched for most clinical characteristics. We identified a higher use of acetylsalicylic acid in pregnant women than in controls. This finding was predicted since this medication is usually prescribed to pregnant women with a history of diabetes to reduce the risk of developing PE^12^.

Our main goal was to characterize circulating Tregs; however, up to the moment, the best markers to define these cells and their subsets were not established, especially regarding CD8+ Tregs lymphocytes. The percentage of these Treg subsets in peripheral blood is very low; thus, it is difficult to investigate, mainly during pregnancy when the sample volume to test needs to be more limited^7^. So far, Pellegrino and colleagues reported a lower percentage of CD8+ Tregs (characterized as CD8+CD25+Foxp3+ Treg) in long-term T1DM patients when compared to healthy controls^13^. In pregnancy, a lower percentage of CD8+ Tregs cells was also described in PE^14^. We identified a lower percentage of CD8+ Treg cells in T1DM pregnant women compared to nonpregnant T1DM women, which suggest that these cells may play a role in the maternal immune response in this clinical condition.

There is no consensus regarding the frequency of circulating CD8 lymphocytes in T1DM patients. We observed an increased percentage of total CD8+ lymphocytes in the peripheral blood of the T1DM pregnant group compared to the nonpregnant patients. Wiedeman et al.^15^ suggested that there is a relationship between TCD8+ exhaustion and the timing of T1DM progression; however, these findings were not confirmed by other investigators^16,17^. In addition, Pellegrino et al.^13^ did not observe quantitative differences in this cell subset between individuals with long-term T1DM and healthy controls. Meggyes et al.^18^ also did not detect significant differences in the frequency of this cell population between healthy pregnant and nonpregnant women. Further investigations are needed to better elucidate the role of TCD8+ in T1DM and in healthy pregnancy.

Groen et al.^19^ detected differences in the percentage of TCD4CD25- and TCD4CD25+FOXP3+ cells between nonpregnant women with and without T1DM, but they did not identify significant changes comparing healthy and T1DM pregnant women. This group suggested that the observed increased frequency of circulating Treg in patients with T1DM could be to compensate for functional deficiencies that these cells seem to present in these cases. In contrast, Amouyal et al.^20^ showed a lower amount of CD4+ Treg in T1DM women during the third trimester when compared with healthy women in the same gestational period. Our analysis was restricted to women with T1DM, pregnant or not; thus, the differences found can be attributed to pregnancy. Lima et al.^21^ identified a reduction in Treg through physiological pregnancy; they also demonstrated lower expression of FOXP3 in CD4lowCD25+high Treg cells in pregnancy, showing a reduction in lymphocytes with suppressive profile.

There is evidence that, due to its modulatory property, an increased number of Treg lymphocytes is relevant for a successful pregnancy^21,22^. Indeed, Green et al.^23^ reported that this change can be observed in both circulation and the decidua during a healthy pregnancy. In turn, other researchers have found greater amounts of these cell subsets in the decidua than in peripheral blood. Furthermore, Keller et al.^24^ showed more phenotypical and quantitative significant changes of Treg subsets in the decidua than in circulation. Our analysis included only the circulating cell population; thus, we hypothesized that the Treg subsets decreased in pregnant women due to cells’ migration to the decidua to prevent obstetric complications.

Our data showed changes in circulating T-cell subsets associated with pregnancy, but in any case, it did not affect the gestational development as only one of the pregnant women developed PE, and all the other cases evolved without obstetric and fetal complications. It is well recognized that other cell populations are also involved in maternal–fetal interactions and in the development of T1DM^25^. The balance between all these elements and the profile of the mediators released are critical for disease control and pregnancy success, and it can be suggested that they have contributed to the observed clinical results.

Our study did not include a nondiabetic pregnant control group because we wanted to see how pregnancy itself changes the pattern of Treg cells. We also wanted to evaluate the successive changes during pregnancy, which is why we evaluated the three trimesters separately. Comparing our data with nondiabetic pregnant women should be a further study.

## CONCLUSION

Taken together, our results stimulate new investigations, including a larger number of participants and groups of healthy pregnant and nonpregnant women. In addition, it is important to perform quantitative and qualitative assays to better characterize these cell subsets and their role in pregnancy in T1DM women. However, we have demonstrated changes in the circulating Treg cell profile that seem to be associated with pregnancy in T1DM patients.
